# Individual and Combined Effects of Black Cumin Seeds and Turmeric Powder on Performance and Egg Quality Traits in Laying Hens

**DOI:** 10.1002/vms3.70639

**Published:** 2025-10-09

**Authors:** Shanaz Alam Sunny, Md. Mufazzal Hossain, Mofassara Akter, Md. Imran Hossain, Md. Ratan

**Affiliations:** ^1^ Department of Animal Nutrition Genetics and Breeding Sher‐e‐Bangla Agricultural University, Sher‐e‐Bangla Nagar Dhaka Bangladesh

**Keywords:** black cumin seed, diet, hen, pullets, supplementation, turmeric powder

## Abstract

**Background:**

Rising feed costs and concerns over synthetic additives have increased interest in natural alternatives to improve poultry productivity.

**Objectives:**

This study investigated the individual and synergistic effects of dietary black cumin seed and turmeric powder supplementation in laying hens.

**Methods:**

Four hundred eighty 19‐week‐old Isa Brown pullets were randomly allocated, in a completely randomised design, to four treatment groups with four replications of 30 birds each and raised from 19 to 30 weeks of age. The experimental diets were formulated as follows: T_0_ (control, without black cumin seed and turmeric powder), T_1_ (1% black cumin seed), T_2_ (1% turmeric powder) and T_3_ (0.5% black cumin seed + 0.5% turmeric powder).

**Results:**

Supplementation with black cumin seed (T_1_) significantly improved hen‐day egg production, egg mass, shape index, shell thickness, Haugh unit, and yolk–albumen ratio compared with the control. Turmeric powder (T_2_) was most effective in enhancing yolk colour and also improved shell weight and albumen quality. Combined supplementation (T_3_) did not show additive effects and, in some traits, resulted in lower values than individual supplementation. Feed conversion ratio was not significantly different among treatments, though T_0_ and T_2_ showed numerically better efficiency than T_1_ and T_3_.

**Conclusion:**

Black cumin seed supplementation (1%) is effective for improving overall egg production and quality, while turmeric powder (1%) is particularly beneficial for yolk pigmentation. Combined supplementation may not be advantageous. These findings highlight the potential of black cumin seed and turmeric powder as natural feed additives to enhance laying performance and egg quality in layer diets.

## Graphical abstract

The experiment investigated dietary supplementation of black cumin seed and turmeric powder in laying hens. Four treatments were applied: control, 1% black cumin, 1% turmeric, and a 0.5% combination. Productive performance, feed conversion ratio, egg quality parameters, and shell characteristics were measured to evaluate the efficacy of these phytogenic additives.

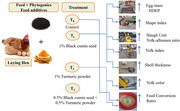

## 1 Introduction

Poultry farming is a fast‐growing sector worldwide, playing a significant role in the economics of countries like Bangladesh, where it contributes about 1.5 to 1.6% of the GDP (Karmoker 2022). Eggs serve as an affordable and nutritious source of protein, vitamins, and other nutrients (Elson et al. 2011; Ukwu et al. 2017), often described as ‘miracle food’ due to their health benefits (Damaziak et al. 2017; Molnár et al. 2020). To meet increasing global demand – reflected by a 24% rise in egg production over the last decade (FAO 2023). Intensive poultry farming practices have become common, though these can introduce stress and reduce productivity (Olobatoke et al. 2011). Historically, antibiotics were added to chicken feed to keep birds healthy and improve egg quality (Steiner 2006). But because of health concerns about antibiotic resistance in humans, many countries now ban their use for growth (Casewell et al. 2003). This has driven interest in natural alternatives such as herbal feed additives, which offer safety, cost‐effectiveness, and health benefits (Diaz‐Sanchez et al. 2015; Alagawany et al. 2021).

Black cumin (*Nigella sativa*) is an herb known for immunostimulatory, antimicrobial, and antioxidant properties, mainly due to thymoquinone (Raheem et al. 2021; Zaky et al. 2021; Manjunath et al. 2020). According to certain research, broiler feeds with 1 g/kg of black cumin seeds may experience an increase in body weight and feed conversion (Erener et al. 2010). When added to laying hen diets, black cumin may protect magnum and uterine cells (Dhama et al. 2015). It may lower cholesterol and increase laying hen productivity at a dose of 3% (Aydin et al. 2008).

Turmeric (*Curcuma longa*) contains curcumin, a potent antioxidant (Sanghvi et al. 2020; Osawa et al. 1995; Rajput et al. 2013), and has demonstrated improved nutrient absorption and yolk colour in poultry (Ammon et al. 1993; Platel and Srinivasan 2000). In broiler chicken diets, curcumin at a level of 0.2 g/kg can increase the length and weight of the duodenum, jejunum, and broiler ceca (Rajput et al. 2013). Nutrient digestibility is improved as a result. Turmeric powder supplementation can improve egg weight, feed conversion, and egg production (Gumus et al. 2018).

While these herbs have individual benefits, their combined effect on laying hen performance remains underexplored. This study evaluates the impact of dietary black cumin seed and turmeric powder, alone and in combination, on productive performance and egg quality traits of laying hens.

## 2 Materials and Methods

### 2.1 Statement of the Experiment

The research work was conducted at the poultry farm of Sher‐e‐Bangla Agricultural University, Dhaka‐1207, using four hundred eighty 19‐week‐old Isa Brown pullets over a period of 84 days, from the 19^th^ week to the 30^th^ week of age (August 2023 to October 2023), to assess the feasibility of incorporating dietary black cumin seeds and turmeric powder into the diets of laying hens on performance and egg quality traits.

### 2.2 Experimental Birds and Management

Four hundred eighty pullets were obtained from a local hatchery, individually weighed, and randomly assigned to four dietary treatments in a cage system, with four replicates per treatment and thirty birds per replicate. The treatments included T_0_ (control), T_1_ (1% black cumin seed), T_2_ (1% turmeric powder), and T_3_ (0.5% black cumin seed + 0.5% turmeric powder). The facility was cleaned with tap water and disinfected using iodophor solution (3 mL/L) before cage installation. Birds were fed a commercial layer diet (Kazi Feed Ltd.) (Table 1) with daily incorporation of feed additives. Fresh water was provided ad libitum. Drinkers and feeders were cleaned weekly and monthly, respectively. A 16‐hour light schedule was maintained, with light intensity recorded twice daily. Temperature and humidity were monitored daily at 10:30 a.m. Strict sanitation protocols were followed to ensure bird health.

**TABLE 1:** The ingredients and chemical composition of the basal diet.

Attributes	Content (%)
**Ingredient composition**	
Maize	50.00
Soybean meal	18.00
Full fat soybean	5.00
Rice polish	10.00
Rice bran	4.50
Rapeseed meal	3.00
Deoiled rice bran	2.00
Vegetable oil	3.00
Limestone powder	2.00
Dicalcium phosphate	1.50
Common salt	0.30
Vitamin‐mineral premix	0.50
Enzyme + probiotic	0.20
**Chemical composition (% DM basis)**	
Dry matter	88
Crude protein	16
Ether extract	4
Crude fibre	5
Methionine	0.41
Lysine	0.80
Metabolisable energy (Kcal/kg)	2750

### 2.3 Egg Production and Quality Assessment

Egg production was recorded daily, with weekly measurements of feed intake and average egg weight. Feed conversion ratio (FCR) was calculated as total feed consumed divided by total egg mass. Hen‐day egg production (HDEP) and egg mass were derived accordingly. Each week, ten eggs per replicate were randomly collected for quality assessment. Egg shape index was calculated from egg length and width using digital callipers. Albumen quality was evaluated using Haugh unit scores based on albumen height and egg weight (Doyon et al. 1986). Broken eggs were assessed for albumen and yolk height using a spherometer. Eggshells were washed, dried, and weighed; yolk and shell weights were recorded, and albumen weight was determined by difference. Shell percentage and yolk‐to‐albumen ratio were calculated. Albumen and yolk indices were determined following Heiman and Carver (1936) and Card and Nesheim (1972), respectively. Yolk colour was assessed using the DSM Yolk Colour Fan, and eggshell thickness was measured at three points using a screw gauge.

### 2.4 Statistical Analysis

Statistical analysis was carried out with Statistical Package for the Social Sciences program 29.0 (SPSS). All data were analysed using one‐way ANOVA. Differences among the groups were calculated using Duncan multiple range test. Level of significance was taken as *P* < 0.05.

## 3 Results and Discussion

### 3.1 Hen Day Egg Production (HDEP)

**FIGURE 1:**
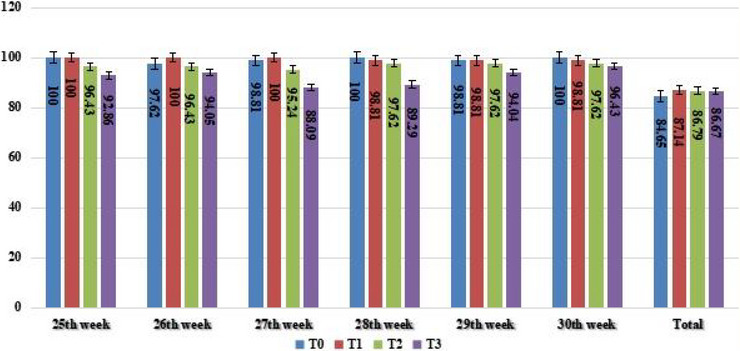
Weekly HDEP performance in different groups.

HDEP of laying hens in different groups was presented weekly in Figure 1. The results indicated that hen‐day egg production peaked around the 25^th^ week, with the T_1_ group showing significantly higher production at week 27 (*P* < 0.05) compared to the T_0_, T_2_ and T_3_ groups. The significantly higher egg production observed in the T_1_ group may be due to the bioactive components of black cumin seed, particularly thymoquinone, which enhance antioxidant status, nutrient absorption, and possibly reproductive hormone activity. These effects collectively support better ovarian function and egg laying. These results align with the findings of Singh et al. (2019), who reported that supplementation with 1% black cumin seed, garlic, and turmeric rhizome powder, either individually or in combination, significantly (*P* < 0.05) improved egg production performance in White Leghorn laying hens. Similarly, Hossain et al. (2016) found a significant effect of different levels of black cumin supplementation on the egg production of laying hens. Conversely, the benefits of turmeric powder supplementation on egg production during the 28–34 weeks of age are also supported by positive findings in the literature (Malekizadeh et al. 2012; Park et al. 2012; Rahardja et al. 2015; Kanagaraju et al. 2017; Johannah et al. 2018; Gumus et al. 2018).

### 3.2 Egg Mass (g/hen/day)

The effect of dietary supplementation of black cumin seed and turmeric powder on egg mass is shown in Table 2. Across the experimental period, birds in the T_0_ (control) and T_1_ groups consistently recorded higher egg mass compared to T_3_ (combination of black cumin seed and turmeric powder), which showed significantly lower egg mass values (*p* < 0.05) in several weeks (25^th^, 27^th^ and 30^th^ weeks). The total mean egg mass was highest in the T_0_ group (54.62 ± 0.89 g/hen/day), followed by T_1_ and T_2_, while T_3_ had the lowest total egg mass (47.95 ± 3.17 g/hen/day). These findings suggest that combining supplementation may negatively influence egg production performance. Similar trends were reported by Riasi et al. (2012) and Laganá et al. (2011), who found no weekly differences in egg mass but noted that moderate supplementation could enhance production. Conversely, these results contradict the findings of Dalal et al. (2018) and Park et al. (2012), who reported that supplementation with turmeric at 0.50% or 1% significantly increased egg mass compared to the control diets.

**TABLE 2:** Effect of dietary black cumin seed and turmeric powder on egg mass (g/hen/day) and feed conversion ratio of laying hen.

Parameter	Treatment	24^th^ week	25^th^ week	26^th^ week	27^th^ week	28^th^ week	29^th^ week	30^th^ week	Total week
**Egg mass**	T_0_	48.65 ± 2.31	54.68^a^ ± 0.55	52.36 ± 2.35	55.23^a^ ± 1.30	56.21^a^ ± 0.59	56.13 ± 0.87	59.07^a^ ± 0.56	54.62^a^ ± 0.89
	T_1_	50.15 ± 1.04	53.23^ab^ ± 0.64	53.37 ± 0.61	54.85^a^ ± 0.24	53.11^ab^ ± 1.88	56.31 ± 1.07	57.09^ab^ ± 1.80	54.02^ab^ ± 0.49
	T_2_	50.25 ± 1.52	52.68^ab^ ± 0.88	53.08 ± 2.60	51.69^ab^ ± 2.83	54.19^ab^ ± 1.71	55.31 ± 2.46	56.69^ab^ ± 1.47	53.42^ab^ ± 1.76
	T_3_	44.98 ± 1.77	46.82^b^ ± 4.21	47.94 ± 4.56	44.28^b^ ± 5.50	46.31^b^ ± 5.32	51.74 ± 5.40	53.55^b^ ± 1.17	47.95^b^ ± 3.17
**FCR**	T_0_	2.23 ± 0.07	2.05 ± 0.02	2.10 ± 0.08	2.01^b^ ± 0.03	2.01 ± 0.02	1.97 ± 0.03	1.89^b^ ± 0.02	2.04 ± 0.03
	T_1_	2.21 ± 0.05	2.11 ± 0.02	2.10 ± 0.02	2.03^b^ ± 0.01	2.06 ± 0.03	2.00 ± 0.03	1.95^ab^ ± 0.04	2.07 ± 0.02
	T_2_	2.18 ± 0.07	2.10 ± 0.05	2.04 ± 0.04	2.07^ab^ ± 0.06	2.05 ± 0.05	1.97 ± 0.06	1.95^ab^ ± 0.02	2.05 ± 0.05
	T_3_	2.31 ± 0.09	2.25 ± 0.12	2.23 ± 0.13	2.28^a^ ± 0.13	2.23 ± 0.12	2.11 ± 0.15	2.02^a^ ± 0.02	2.20 ± 0.09

Values expressed as mean and standard error (SE). ^a,b^different superscript letters in a column denote significant differences at *p* < 0.05 level.

### 3.3 FCR

The FCR remained statistically similar across all groups during most weeks (24, 25, 26, 28, and 29) (Table 2). However, in weeks 27 and 30, the T_3_ and T_1_ groups had significantly higher FCR values (*P* < 0.05), indicating reduced feed efficiency compared to the control and T_2_ treatment groups. The total average FCR over the 7‐week period was also highest in the T_3_ and T_1_ groups, further supporting the observation of reduced feed efficiency with higher supplementation levels. These results are consistent with those reported by Yalçin et al. (2012), who observed a significant increase in FCR in laying hens upon black cumin supplementation. Similarly, Abou‐Elkhair et al. (2018) also reported that dietary inclusion of phytogenic feed additives (fennel, black cumin or hot red pepper) led to significant (*P* < 0.05) differences in FCR compared with the non‐supplemented control group throughout the experimental period (32 to 40 weeks of age). However, the present results contradict the findings of Suwarta et al. (2022), who reported no significant differences in FCR among treatments T_1_ (2.5 g TP + 2.5 g BCP/kg ration), T_2_ (5 g TP + 5 g BCP/kg ration), and T_3_ (7.5 g TP + 7.5 g BCP/kg ration), with all treatment groups showing significantly lower FCR values than the control (T_0_). In their study, the lowest FCR was observed in T_3_ (3.59), whereas the highest was recorded in T_0_ (3.88).

### 3.4 External Quality of Eggs

The external egg quality parameters—shape index, shell weight, shell thickness, and shell percentage—were influenced by both treatment and week of collection, with notable variations observed throughout the study period.

### 3.5 Shape Index

Shape index values fluctuated slightly across weeks and treatments. At week 29, T_1_ exhibited a significantly (*p* < 0.05) higher shape index compared to T_3_, while the values for T_0_ and T_2_ remained intermediate (Table 3). Over the total experimental period, the shape index ranged from 78.03 (T_3_) to 80.29 (T_1_). These findings are in alignment with those reported by Malekizadeh et al. (2012), who observed the highest numerical value of egg shape index in the group supplemented with 1% *Curcuma longa*. Similarly, Singh et al. (2019) reported no significant differences in egg shape index among different treatment groups. In their study, the treatment groups included a basal diet with an antibiotic (Enramycin at 2.5 g per quintal of feed), a feed additive (T_2_), 1% black cumin powder (T_3_), 1% garlic powder (T_4_), 1% turmeric powder (T_5_), 1% mixture of black cumin and turmeric in equal proportion (T_6_), another 1% mixture of black cumin and turmeric (T_7_), 1% mixture of garlic and turmeric in equal proportion (T_8_) and 1% mixture of black cumin seed, garlic and turmeric in equal proportion (T_9_).

**TABLE 3:** Effect of dietary black cumin seed and turmeric powder on external quality traits of eggs.

Parameter	Treatment	24^th^ week	25^th^ week	26^th^ week	27^th^ week	28^th^ week	29^th^ week	30^th^ week	Total week
**Shape index**	T_0_	77.88 ± 1.76	79.05 ± 0.82	79.18 ± 1.10	78.35 ± 1.16	78.84 ± 1.04	78.20^ab^ ± 1.42	78.46 ± 0.72	78.57 ± 0.84
	T_1_	78.85 ± 1.17	78.94 ± 1.22	80.54 ± 0.58	79.68 ± 0.55	79.33 ± 0.39	84.15^a^ ± 3.80	80.54 ± 0.13	80.29 ± 0.27
	T_2_	80.86 ± 0.41	76.44 ± 1.86	78.14 ± 1.73	79.82 ± 0.70	78.33 ± 0.81	80.13^ab^ ± 1.06	76.98 ± 4.04	78.67 ± 0.97
	T_3_	79.62 ± 0.89	79.37 ± 1.13	79.90 ± 0.94	76.73 ± 1.93	75.31 ± 2.69	75.25^b^ ± 2.17	80.04 ± 0.50	78.03 ± 0.47
**Shell weight**	T_0_	5.28^b^ ± 0.11	5.79 ± 0.16	5.61^c^ ± 0.43	6.68 ± 0.17	6.23 ± 0.13	6.09 ± 0.23	6.57 ± 0.24	6.04 ± 0.07
	T_1_	5.56^ab^ ± 0.09	5.66 ± 0.17	6.97^ab^ ± 0.10	6.62 ± 0.11	7.09 ± 0.18	6.09 ± 0.26	5.94 ± 0.12	6.28 ± 0.07
	T_2_	5.95^a^ ± 0.24	6.22 ± 0.26	7.34^a^ ± 0.41	6.46 ± 0.12	6.82 ± 0.38	5.85 ± 0.20	6.11 ± 0.35	6.40 ± 0.80
	T_3_	5.53^ab^ ± 0.18	5.78 ± 0.28	6.16^bc^ ± 0.24	6.30 ± 0.28	6.99 ± 0.29	6.56 ± 0.21	6.17 ± 0.29	6.22 ± 0.18
**Shell thickness**	T_0_	0.35^b^ ± 0.01	0.37 ± 0.01	0.32^b^ ± 0.02	0.36^b^ ± 0.00	0.34^b^ ± 0.01	0.36 ± 0.00	0.37 ± 0.00	0.36^b^ ± 0.00
	T_1_	0.35^b^ ± 0.01	0.36 ± 0.01	0.39^a^ ± 0.01	0.40^a^ ± 0.01	0.38^a^ ± 0.01	0.36 ± 0.01	0.36 ± 0.00	0.38^a^ ± 0.01
	T_2_	0.40^a^ ± 0.01	0.37 ± 0.01	0.37^a^ ± 0.01	0.37^ab^ ± 0.01	0.38^a^ ± 0.01	0.36 ± 0.00	0.36 ± 0.01	0.38^a^ ± 0.00
	T_3_	0.37^ab^ ± 0.01	0.38 ± 0.01	0.36^ab^ ± 0.01	0.36^b^ ± 0.01	0.37^ab^ ± 0.01	0.38 ± 0.00	0.36 ± 0.00	0.37^ab^ ± 0.01
**Shell per cent**	T_0_	9.74^b^ ± 0.22	10.51 ± 0.30	10.12^b^ ± 0.79	11.76 ± 0.31	11.03^b^ ± 0.23	10.37^ab^ ± 0.40	11.25 ± 0.41	10.69^b^ ± 0.12
	T_1_	10.69^ab^ ± 0.15	11.24 ± 0.33	12.69^a^ ± 0.18	11.96±0.21	12.73^a^±0.33	10.38^ab^±0.45	10.40±0.22	11.44^a^±0.13
	T_2_	10.97^a^ ± 0.46	11.09 ± 0.47	12.93^a^ ± 0.73	11.37 ± 0.21	11.82^ab^ ± 0.65	10.05^b^ ± 0.36	10.15 ± 0.58	11.20^ab^ ± 0.15
	T_3_	10.57^ab^ ± 0.35	10.72 ± 0.52	11.24^ab^ ± 0.43	11.48 ± 0.51	12.52^ab^ ± 0.53	11.41^a^ ± 0.36	10.49 ± 0.50	11.21^ab^ ± 0.12

Values expressed as mean and standard error (SE). ^a,b^different superscript letters in a column denote significant differences at *p* < 0.05 level.

### 3.6 Shell Weight

Shell weight showed consistent treatment‐related differences. T_2_ recorded the highest shell weight at week 26, significantly (*p* < 0.05) greater than T_0_, with T_1_ also showing a similar peak at the same week (Table 3). In contrast, T_0_ consistently yielded lower shell weights, particularly at week 24. By week 30, shell weights stabilised across treatments, ranging between 5.94 and 6.57 g. The total mean shell weight ranged from 6.04 g (T_0_) to 6.40 g (T_2_). These results are consistent with those of Attia et al. (2014), who reported similar values for shell weight. Additionally, Malekizadeh et al. (2012) found that the highest numerical value for shell weight was observed in eggs from hens supplemented with 1% *Curcuma longa*. Conversely, Dalal et al. (2018) reported that feeding laying hens various levels of turmeric powder (0.0, 0.50, 1.0, 1.5 and 2.0 g/kg of feed) had no significant effect on eggshell weight or the shell weight to egg weight ratio.

### 3.7 Shell Thickness

Shell thickness was significantly affected by treatment at several time points. At week 26, T_1_ exhibited the thickest shells, significantly (*p* < 0.05) thicker than T_0_ (Table 3). A similar trend was observed at weeks 27 and 28, where T_1_ and T_2_ maintained higher shell thickness values compared to T_0_ and T_3_. Across all treatments and weeks, shell thickness values remained within a narrow range (0.32–0.40 mm), indicating moderate variation. The overall mean shell thickness ranged from 0.36 mm in T_0_ to 0.38 mm in T_1_ and T_2_ (*P* < 0.05). These findings are not in alignment with those of El Bagir et al. (2006); Yalçin et al. (2012), who reported non‐significant (*P* > 0.05) differences in shell weight, shell thickness, and shell breaking strength due to black cumin supplementation in laying hens. Similarly, non‐significant differences in shell thickness and shell strength due to garlic supplementation (Canogullari et al. 2010; Kaya et al. 2012; Dalal et al. 2018) and turmeric supplementation (Laganá et al. 2011; Park et al. 2012) on eggshell quality have been reported.

### 3.8 Shell Per cent

Shell per cent demonstrated clear differences among treatments, particularly at weeks 26 and 28 (Table 3). At week 26, T_2_ and T_1_ recorded the highest shell percentages, significantly (*p* < 0.05) surpassing T_0_. At week 28, T_1_ maintained a significantly (*p* < 0.05) higher percentage compared to T_0_. T_3_ also showed strong performance in later weeks, particularly at week 29. Over the total experimental period, T_1_ maintained the highest average shell percentage (11.44%), while T_0_ had the lowest (10.69%). These results are consistent with those of Malekizadeh et al. (2012), who reported that the highest numerical value for shell weight was observed in eggs from hens supplemented with 1% *Curcuma longa*. Conversely, Dalal et al. (2018) found that feeding laying hens various levels of turmeric powder (0.0, 0.5, 1.0, 1.5, and 2.0 g/kg of feed) had no significant (*p* > 0.05) effect on eggshell weight or the shell weight‐to‐egg weight ratio.

### 3.9 Internal Quality of Eggs

The internal quality characteristics of eggs—Haugh unit, albumen index, yolk index, yolk‐to‐albumen ratio, and yolk colour grading—were significantly influenced by the treatments across the observed weeks.

### 3.10 Haugh Unit

Haugh unit values varied across treatments and weeks, with T_2_ exhibiting the highest value at week 24 (Table 4). Although fluctuations were found across weeks, notable differences emerged at week 28, where T_3_ significantly (*p* < 0.05) outperformed T_0_ and T_1_. By week 29, T_1_ maintained a significantly higher (*p* < 0.05) Haugh unit compared to T_3_. The total mean Haugh unit ranged from 89.35 (T_3_) to 94.27 (T_1_). These findings are consistent with those of Singh and Kumar (2018), who reported a significant (*P* < 0.05) effect on albumen height and Haugh unit due to the incorporation of black cumin, garlic, and turmeric in the layer ration. Significant improvements in Haugh unit due to black cumin supplementation were also reported by Khana et al. (2013) and Chongtham et al. (2015) in laying hens. However, turmeric powder supplementation and ration quality did not significantly affect Haugh unit values, as reported by Saraswati et al. (2013a), Saraswati et al. (2013).

**TABLE 4:** Effects of dietary black cumin seed and turmeric powder on internal quality traits of eggs.

Parameter	Treatment	24^th^ week	25^th^ week	26^th^ week	27^th^ week	28^th^ week	29^th^ week	30^th^ week	Total week
**Haugh unit (score)**	T_0_	96.97 ± 3.89	89.74 ± 4.72	99.40 ± 2.68	97.93 ± 1.96	91.58^b^ ± 1.83	92.25^ab^ ± 3.42	84.00 ± 3.15	93.13 ± 0.83
	T_1_	99.20 ± 8.13	93.75 ± 3.09	99.40 ± 2.68	90.88 ± 4.71	92.86^b^ ± 4.24	94.42^a^ ± 3.13	89.35 ± 3.35	94.27 ± 1.92
	T_2_	102.24 ± 5.47	94.24 ± 2.64	95.84 ± 3.36	89.76 ± 5.09	97.90^ab^ ± 1.12	90.85^ab^ ± 1.83	74.96 ± 6.66	92.26 ± 1.78
	T_3_	92.88 ± 3.65	84.21 ± 3.27	94.49 ± 2.94	86.39 ± 6.03	103.06^a^ ± 0.63	82.07^b^ ± 5.45	82.35 ± 7.72	89.35 ± 2.02
**Albumen index (%)**	T_0_	13.50 ± 1.53	10.61 ± 1.05	14.03 ± 1.09	13.98 ± 0.84	12.43^b^ ± 0.93	11.13^ab^ ± 0.93	10.18 ± 0.57	12.26 ± 0.23
	T_1_	14.27 ± 2.78	11.72 ± 1.21	12.37 ± 0.58	11.37 ± 1.04	12.43^b^ ± 1.67	12.55^a^ ± 0.91	11.25 ± 1.06	12.27 ± 0.63
	T_2_	15.79 ± 2.14	11.56 ± 0.84	12.80 ± 0.78	10.82 ± 1.43	14.09^ab^ ± 0.54	11.13^ab^ ± 0.62	8.28 ± 1.20	12.07 ± 0.44
	T_3_	12.02 ± 1.19	9.69 ± 0.83	12.39 ± 1.27	10.74 ± 1.90	16.08^a^ ± 0.53	9.22^b^ ± 1.34	10.24 ± 1.65	11.48 ± 0.66
**Yolk index (%)**	T_0_	45.60 ± 0.86	44.99^a^ ± 2.07	47.18 ± 1.62	46.69 ± 0.51	44.81 ± 1.57	42.86 ± 2.02	39.60 ± 2.68	44.53 ± 1.20
	T_1_	48.11 ± 0.86	47.62^a^ ± 0.99	48.22 ± 3.52	44.79 ±1.92	47.26 ± 1.44	44.01 ± 1.88	41.68 ± 1.17	45.96 ± 0.24
	T_2_	46.58 ± 1.78	48.77^a^ ± 1.49	46.08 ± 0.98	42.92 ± 1.85	48.10 ± 1.15	43.35 ± 1.24	41.73 ± 1.44	45.36 ± 0.39
	T_3_	47.39 ± 1.22	40.56^b^ ± 0.70	46.58 ± 2.15	42.29 ± 1.33	47.75 ± 2.08	42.98 ± 2.07	42.43 ± 1.55	44.28 ± 1.16
**Yolk‐Albumen ratio**	T_0_	0.33 ± 0.01	0.36 ± 0.03	0.37 ± 0.01	0.36^b^ ± 0.01	0.40 ± 0.02	0.47 ± 0.03	0.42^ab^ ± 0.01	0.39 ± 0.01
	T_1_	0.30 ± 0.03	0.38 ± 0.02	0.43 ± 0.02	0.46^a^ ± 0.02	0.44 ± 0.03	0.45 ± 0.01	0.46^a^ ± 0.01	0.42 ± 0.01
	T_2_	0.34 ± 0.02	0.35 ± 0.01	0.39 ± 0.01	0.40^ab^ ± 0.03	0.42 ± 0.02	0.40 ± 0.01	0.42^ab^ ± 0.01	0.39 ± 0.02
	T_3_	0.35 ± 0.02	0.42 ± 0.01	0.39 ± 0.01	0.38^ab^ ± 0.01	0.38 ± 0.01	0.43 ± 0.02	0.41^b^ ± 0.01	0.40 ± 0.00
**Yolk colour grading**	T_0_	5.50 ± 0.95	5.50 ± 0.64	5.25^b^ ± 0.25	6.25^b^ ± 0.62	7.00 ± 0.40	6.00 ± 0.57	5.00^b^ ± 0.70	5.79^b^ ± 0.41
	T_1_	5.50 ± 0.86	6.25 ± 0.47	5.75^ab^ ± 0.25	7.50^ab^ ± 0.28	7.25 ± 0.47	6.75 ± 0.25	7.00^a^ ± 0.40	6.57^a^ ± 0.18
	T_2_	6.75 ± 0.47	5.50 ± 0.50	6.75^a^ ± 0.47	7.75^a^ ± 0.25	7.50 ± 0.28	7.25 ± 0.25	7.00^a^ ± 0.00	6.92^a^ ± 0.07
	T_3_	5.50 ± 0.64	5.75 ± 0.47	6.25^ab^ ± 0.47	7.25^ab^ ± 0.47	7.75 ± 0.25	7.00 ± 0.40	7.00^a^ ± 40	6.64^a^ ± 0.04

Values expressed as mean and standard error (SE). ^a,b^different superscript letters in a column denote significant differences at *p* < 0.05 level.

### 3.11 Albumen Index

Similar trends were found in albumen index values. T_2_ consistently recorded higher scores, peaking at week 24, while T_3_ achieved the highest value at week 28, significantly (*p* < 0.05) exceeding T_0_ and T_1_ (Table 4). However, by week 29, T_3_ declined significantly, recording the lowest albumen index among treatments. The overall mean was highest in T_1_ (12.27%) and lowest in T_3_ (11.48%). These findings are similar to those reported by Singh et al. (2019), who observed that the egg albumen quality of laying hens differed significantly (*P* < 0.05) following the dietary addition of different herbal additives. Improvements in albumen quality due to supplementation with black cumin seed were also reported by El Bagir et al. (2006) and Yalçin et al. (2012), as well as with turmeric supplementation by Laganá et al. (2011) and Park et al. (2012). Conversely, Dalal et al. (2018) reported that supplementation of turmeric powder had no significant effect on external and internal egg quality parameters in laying hens compared to the control groups.

### 3.12 Yolk Index

Yolk index measurements revealed more stability across treatments, although some significant differences were found. At week 25, T_0_, T_1_, and T_2_ exhibited significantly (*p* < 0.05) higher yolk index values compared to T_3_, with T_2_ reaching the peak value (Table 4). Across the total period, yolk index values ranged from 44.28% (T_3_) to 45.96% (T_1_), showing minimal variation. Phuoc et al. (2019) and Laganá et al. (2019) also reported no significant effect of turmeric supplementation on yolk index and yolk colour, findings that are similar to the present study.

### 3.13 Yolk‐Albumen Ratio

The yolk‐albumen ratio increased progressively with age, with notable differences observed at week 27 (Table 4). T_1_ showed the highest value, significantly (*p* < 0.05) exceeding T_0_. Although T_0_ recorded the highest ratio at week 29, the overall differences across treatments were less pronounced. Over the whole study, T_1_ also showed the highest mean ratio (0.42), whereas T_0_ and T_2_ had lower averages (0.39). These findings are consistent with those of Congjiao et al. (2019). Similarly, Singh et al. (2019) reported no significant effects of black cumin, garlic, and turmeric powder supplementation on yolk weight and yolk percentage. Olobatoke and Mulugeta (2011) and Deko et al. (2018) also found no significant (*P* > 0.05) effect of garlic powder supplementation on yolk weight, yolk percentage, and yolk colour. In addition, Abou‐Elkhair et al. (2018) reported that the addition of hot red pepper or black cumin to the diets of laying hens had no significant effect on yolk weight percentage.

### 3.14 Yolk Colour Grading

Yolk colour scores improved progressively across treatments and weeks. T_2_ consistently produced higher yolk colour grades, particularly at weeks 26 and 28 (Table 4). By week 30, T_1_, T_2_, and T_3_ all recorded significantly (*p* < 0.05) higher scores compared to T_0_, indicating enhanced yolk pigmentation under these treatments. However, the overall mean yolk colour score was significantly (*p* < 0.05) lowest in T_0_ (5.79) compared to the T_2_, T_3_ and T_1_ groups. The present findings are consistent with those of Park et al. (2012), who reported that yolk colour in the group fed 0.50% turmeric powder was significantly higher than in the control group (*P* < 0.05). Singh et al. (2019) also reported that while dietary treatments had no significant effect on egg yolk weight, height, and yolk index, yolk colour values were significantly (*p* < 0.05) affected by the supplementation of turmeric powder alone or in combination with other additives. Conversely, Phuoc et al. (2019) and Laganá et al. (2019) found no significant effect of turmeric supplementation on yolk index and yolk colour.

## 4 Conclusion

Supplementation of laying hen diets with black cumin seed (*Nigella sativa*) and turmeric powder (*Curcuma longa*) can enhance egg production and quality, though effects vary with dosage and combination. The T1 group (1% black cumin seed) showed a significant increase in hen‐day egg production at week 27. However, higher supplementation levels in the T3 group led to reduced egg mass and feed efficiency, suggesting that synergistic inclusion may negatively impact performance. Black cumin seed (1%) improved egg shape index, shell thickness, Haugh unit, and yolk–albumen ratio, indicating overall enhancement of structural and internal egg quality. Turmeric powder (1%) was most effective in improving yolk colour pigmentation and also supported shell weight and albumen quality. In contrast, combined supplementation (0.5% black cumin + 0.5% turmeric) did not produce additive benefits and, in some parameters, performed less favourably than individual supplements.

Overall, the results suggest that black cumin seed is a promising natural feed additive for improving egg quality traits, while turmeric powder is particularly effective for enhancing yolk colour. The choice of supplementation should therefore depend on production goals—structural and internal egg quality improvement with black cumin, or pigmentation enhancement with turmeric.

## Author Contributions

Conceptualisation: Md. Mufazzal Hossain and Shanaz Alam Sunny. Methodology: Shanaz Alam Sunny and Md. Mufazzal Hossain. Investigation: All authors. Writing –Original draft: Shanaz Alam Sunny. Writing – Review and editing: Shanaz Alam Sunny, Md. Mufazzal Hossain and Mofassara Akter. Supervision: Md. Mufazzal Hossain. Data Curation and Analysis: All authors. Validation: All authors. All authors have read and approved the final manuscript.

## Ethics Statement

This study involved only non‐invasive procedures on laying hens, such as monitoring production performance and egg quality traits. No birds were harmed, slaughtered, or subjected to invasive techniques. The hens were maintained under standard husbandry practices during the 11‐week trial period.

## Conflicts of Interest

The authors declare no conflicts of interest.

## Peer Review

The peer review history for this article is available at https://www.webofscience.com/api/gateway/wos/peer‐review/10.1002/vms3.70639.

## Data Availability

Data will be available on request.
